# Annotations

**Published:** 1900-06-16

**Authors:** 


### Annotations.

### The Chinese Question.

The rights of man are curiously limited by his colour—especially in the United States, where, if anywhere, one would expect to find that all men were equal. We do not here refer to the negro; the yellow skin is now worse off. Indeed, if one may accept the measures recently taken in San Francisco for the control of plague as indicating the general American view on the colour question, it would seem as if the Chinese had no rights whatever. It appears that there are 23,000 Chinese in San Francisco, and that Surgeon-General Wyman, of the Marine Hospital Service, insists on having every one of them inoculated with the Haffkine prophylactic. This they do not like, and to stimulate their distaste an organisation of Celestials in that city has threatened to assassinate any Chinaman who submits to the process. These unfortunates thus find themselves most effectually pinned between the devil and the deep sea, and for this and other reasons they show a considerable desire to "trek" into other parts of the Union. To prevent this, however, the officer who has charge of the Federal sanitary interests in the State of California has placed inspectors on the railways to turn them back and prevent Asiatics crossing the State line. The problem of colour is every year becoming more interesting, especially in great countries like the States wherein all nations meet.

### Typhoid Fever in Paris.

To the would-be visitor to Paris it is by no means encouraging to hear of the continued and increasing prevalence of typhoid fever in that city, and the facts are all the more serious in that there appears much reason for believing that it is due to an infection of the water derived from the river Vanne, which has been specially brought to Paris from a long distance in order to furnish the Parisians with a purer supply than the old one which was derived from the Seine. According to *Public Health* there has not since 1892 been a mortality from typhoid fever at all approaching that in the year 1899, when 4,329 cases were reported, as compared with an average annual number during the four preceding years of 1,315. Nor must we go entirely by the number reported during whole years. The time of year at which the cases arise is a very important point in framing an estimate of the gravity of the condition. The natural tendency is for typhoid to prevail principally in the autumn, and we see an illustration of this in the fact that last year, although the number of cases notified in Paris amounted to 4,329, only 398 of these occurred during the first ten weeks of the year. During the first ten weeks of this year, however, no less than 891 cases have been returned, or a good deal over twice as many as in 1899, and if the autumnal rise should this year bear anything like the same numerical relation to the number occurring during the early weeks that it did last year Paris will evidently be in for a veritable epidemic. That it is the Vanne water which is chiefly in fault seems pretty clear from the reports of several competent observers, notwithstanding the efforts being made in official quarters to put a less serious aspect on the affair. It is stated that the specific bacillus has been recognised and isolated from the water taken from the river itself, from a reservoir at Montsouris, and from the supply of two barracks. Not that we should consider this the most important evidence against the water. The sort of evidence we look for in such a case, evidence which, when it is found, almost inevitably condemns a water supply, is a correspondence between the distribution of the disease and that of the water, and this is what seems to be shown in the case of the water derived from the Vanne. Thus in that part of the town of Sens which is supplied with water by a branch taken from the main aqueduct leading from the Vanne typhoid fever has appeared, while other parts with a different supply have escaped, and in Paris itself the same thing has been seen. During February the Sixteenth Arrondissement, which derives its supply solely from the Avre, had not a case, while the Sixth, Seventh, and Eleventh Arrondissements, which are supplied from the Vanne, had 48, 23, and 59 cases respectively.

### Heat Stroke.

The considerable number of cases of heat stroke, resulting in several instances in death, which occurred during the manœuvres at Aldershot last Monday, draws attention again to the old and perennial question of the clothing of our troops. We fear that "weediness" among some, and want of condition among others may have had something to do with the collapse, but there can be but little doubt that much of the responsibility for these unfortunate accidents lies at the door of the tailor. With the soldier, the question of clothing is complicated, and indeed is dominated, by the fact that each individual must when he leaves his barracks be prepared for all the emergencies of the day. We have no sympathy then with the scornful critic, who, pointing to a soldier on parade, asks if that is the sort of costume in which one would care to play football or to run a race. Such criticism is beside the mark. The soldier must be ready to do all sorts of things; he must be able at times to run like a sprinter, and at other times to lie patiently on mother earth perhaps for hours together. It is nonsense then to ask that soldiers, because they have now and again to make violent exertion, should be lightly clad like boating men, who as soon as ever their work is over tumble into their wraps. The clothing of a soldier must of necessity always be a compromise, and may perhaps never be the actual best for any one of the varied circumstances of his life. Still, in view of the fact that variety is the very essence of what has to be provided for, it does seem reasonable to expect that the clothing of the soldier should be capable of variation, and it is here that the military tailor fails. As everyone knows, it is perfectly possible by the use of belts and buttons and other devices to greatly vary the heat-retaining power of one's clothes without taking anything off or putting anything on. Unfortunately this is just what is not contemplated by the military tailor. The great fault of the clothing of our soldiers lies in the fact that it is so designed as to look disreputable unless it is fully buttoned. There is no adaptability about it, and adaptability should be its very essence. Actual warfare teaches many things, and even army tailors have shown that they can produce proper clothing when it is asked for. Let us ask for it. Aldershot is no child's play, and the more it becomes a place for the teaching of the art of war the more should the conditions of those engaged there conform to those of actual warfare.

